# Supplementary material for: Cryo-EM structure of native human thyroglobulin
Source: Nat Commun. 2022 Jan 10;13:61. doi: 10.1038/s41467-021-27693-8 (PMC8748809; doi:10.1038/s41467-021-27693-8)
Supplement: Supplementary file 1 — Supplementary Information [file 41467_2021_27693_MOESM1_ESM.pdf]

Supplementary Information

Cryo-EM structure of native human thyroglobulin

Ricardo Adaixo[1*], Eva M. Steiner[2*], Ricardo D. Righetto[1*], Alexander Schmidt[3], Henning Stahlberg[1,4#] and Nicholas M. I. Taylor[2#]

[1] Center for Cellular Imaging and NanoAnalytics, Biozentrum, University of Basel, Mattenstrasse 26, CH-4058 Basel, Switzerland

[2] Novo Nordisk Foundation Center for Protein Research, University of Copenhagen, Blegdamsvej 3B, DK-2200 Copenhagen, Denmark

[3] Proteomics Core Facility, Biozentrum, University of Basel, Klingelbergstrasse 72, CH-4058 Basel, Switzerland

[4] current address: Lab. of Biol. Electron Microscopy, Institute of Physics, SB, EPFL, and Dep. of Fund. Microbiol., Faculty of Biology and Medicine, UNIL, CH-1015 Lausanne, Switzerland, Switzerland

* These authors contributed equally

[#] **Corresponding authors:**

Nicholas M. I. Taylor
Novo Nordisk Foundation Center for Protein Research
University of Copenhagen
Blegdamsvej 3B
DK-2200 Copenhagen N, Denmark
Phone: +45 353 35337
E-mail: nicholas.taylor@cpr.ku.dk

Henning Stahlberg
Laboratory of Biological Electron Microscopy
Institute of Physics, SB, EPFL
CH-1015 Lausanne, Switzerland
Phone: +41 21 693 45 07
E-mail: henning.stahlberg@epfl.ch

Supplementary Table 1. Cryo-EM data collection and image processing summary.

**Cryo-EM**	**EMD-12073**
Microscope	Titan Krios
Voltage [kV]	300
Direct electron detector	Gatan K2
Zero-loss energy filter	GIF (20 eV slit width)
Physical pixel size [Å]	0.64
Super-resolution mode	No
Total exposure [e^{-}/Å^{2}]	50
Exposure time [s]	10
Frames per movie	50
Defocus range [µm]	0.5—2.5
Movies acquired	8,119
Beam-image shift	7 shots per hole
Movies processed	7,266
Pixel size [Å]	0.64
Box size [pixels²]	512
Particles picked (Gautomatch w/ templates)	593,563
Particles after 2D classification	99,184
Particles after 3D classification	37,619
Consensus map resolution [Å] (FSC 0.143)	3.3
Composite map resolution [Å] (FSC 0.143)	3.2
**Model**	**PDB 7B75**
Model-to-map fit (composite)	
CC_mask	0.7846
CC_volume	0.7763
CC_peaks	0.7120
Model-to-map resolution (composite) [Å] (FSC 0.5)	3.3
MolProbity statistics	
All-atom clashscore	5.02
Ramachandran plot	
Outliers [%]	0.02
Allowed [%]	8.63
Favored [%]	91.34
Rotamer outliers [%]	0.07
Cbeta deviations [%]	0.00
Peptide plane [%]	0.00
Cis-proline [%]	0.00
Cis-general [%]	0.00
Twisted proline [%]	0.00
Twisted general [%]	0.04
MolProbity score	1.77

Supplementary Table 2. Comparing hTG domain names and borders.

Human Thyroglobulin Domain Borders*			
**No.**	**Domain Area & Description Classical**	**Domain Area & Description hTG structure Coscia *et al.* 2020**	**Domain Area & Description hTG structure This study**
1	1 – 19 Signal Peptide		
2	31 – 92 TG type-1 1	31 – 92 TG type-1 A-domain – NTD	30 – 92 hTG type-1 repeat 1
3	93 – 160 TG type-1 2	93 – 160 TG type-1 B-domain – NTD	93 – 160 hTG type-1 repeat 2
4	161 – 297 TG type-1 3	161 – 297 TG type-1 C-domain – NTD	161 – 297 hTG type-1 repeat 3 (NHI-3a,b 198 – 232, 254 – 293)
5	298 – 358 TG type-1 4	298 – 358 TG type-1 D-domain – NTD	298 – 358 hTG type-1 repeat 4
6		359 – 620 Helical E-domain – NTD	359 – 658 hTG type-1 repeat 5 (NHI-5, 378 – 615)
7	521 – 545 Disordered		
8	605 – 658 TG type-1 5	621 – 658 TG type-1 F-domain – Core	
9	659 – 726 TG type-1 6	659 – 726 TG type-1 G-domain – Core	659 – 726 hTG type-1 repeat 6
10	727 – 921 TG type-1 7	727 – 921 TG type-1 H-domain – Core	727 – 921 hTG type-1 repeat 7 (NHI-7, 777 – 894)
11	922 – 1073 TG type-1 8	922 – 1008 Similar TG type-1 (dimer) I-domain – Core	922 – 1073 hTG type-1 repeat 8 (NHI-8, 937 – 1026)
12		1009 – 1022 Spacer - Core	
13		1023 – 1073 TG type-1 J-domain – Core	
14	1074 – 1145 TG type-1 9	1074 – 1145 TG type-1 K-domain – Core	1074 – 1145 hTG type-1 repeat 9
15	1146 – 1210 TG type-1 10	1146 – 1211 TG type-1 L-domain – Core	1146 – 1270 hTG type-1 repeat 10
16		1210 – 1283 Ig-like domain M-domain – Flap	
17		1284 – 1438 Ig-like domain N-domain – Flap	
18	1456 – 1469 TG type-2	1439 – 1510 TNF/EGF/laminin-like O-domain – Arm	1456 – 1469 hTG type-2 repeat 1
19	1470 – 1486 TG type-2		1470 – 1486 hTG type-2 repeat 2
20	1487 – 1503 TG type-2		1487 – 1503 hTG type-2 repeat 3
21	1511 – 1565 TG type-1 11	1511 – 1565 TG type-1 P-domain - Arm	1511 – 1565 hTG type-1 repeat 11
22	1603 – 1723 Type IIIA	1603 – 1723 TG type-3 Q-domain - Arm	1598 – 1723 hTG type-3a repeat 1
23	1724 – 1892 Type IIIB	1724 – 1892 TG type-3 R-domain - Arm	1724 – 1892 hTG type-3b repeat 2
24	1893 – 1995 Type IIIA	1893 – 1995 TG type-3 S-domain - Arm	1893 – 1995 hTG type-3a repeat 2
25	1996 – 2129 Type IIIB	1996 – 2129 TG type-3 T-domain - Arm	1996 – 2129 hTG type-3b repeat 2
26	2130 – 2187 Type IIIA	2130 – 2186 TG type-3 U-domain - Arm	2130 – 2187 hTG type-3a repeat 3
27	2188 – 2728	2187 – 2728	2188 – 2727

	ChEL	CTD V-domain ChEL (dimer)	ChEL
28	2729 – 2768<br>Disordered		

*… The amino acid numbering system presented is including signal peptide residues 1 – 19, following Uniprot: P01266 numbering
NTD … N-terminal domain, residues 20 – 620
NHI … Non-homologous insertions
Core … residues 621 – 1210
Flap … residues 1211 – 1438
Arm … residues 1439 – 2186
CTD … C-terminal domain, Arche-like, residues 2187 – 2768
ChEL … Cholinesterase -like domain

Supplementary Table 3. Cysteines and opposing cysteine pairs in the hTG structure.

Pair #	Cysteine Residues*	Structural DSB Distance (Å)	Domain	Comment
	C15	N.A.		Not modeled
1	C34 – C52	2.03	30 – 92<br>hTG type-1 repeat 1	Disulfide bond present in structure
2	C63 – C70**	2.03		
3	C72 – C92	2.02		
4	C96 – C120	2.03	93 – 160<br>hTG type-1 repeat 2	
5	C131 – C138**	2.03		
6	C140 – C160	2.03		
7	C164 – C183	2.03	161 – 297<br>hTG type-1 repeat 3	
8	C194 – C235	2.03		
9	C237 – C297	2.03		
10	C301 – C319	2.03	298 – 358<br>hTG type-1 repeat 4	
11	C330 – C336**	2.03		
12	C338 – C358	2.03		
13	C364 – C620	2.03	359 – 658<br>hTG type-1 repeat 5	
14	C408 – C608	2.03		
15	C631 – C636	2.03		
16	C638 – C658	2.03		
17	C662 – C687	2.03	659 – 726<br>hTG type-1 repeat 6	
18	C698 – C703	2.03		
19	C705 – C726	2.02		
20	C730 – C763	2.02	727 – 921<br>hTG type-1 repeat 7<br>(NHI-7, 777 – 894)	
21	C774 – C898**	2.03		
22	C900 – C921	2.03		
23	C925 – C1031	2.03	922 – 1073<br>hTG type-1 repeat 8<br>(NHI-8, 937 – 1026)	
24	C1042 – C1049	2.03		
25	C1051 – C1073	2.02		
26	C1077 – C1108	2.03	1074 – 1145<br>hTG type-1 repeat 9	
27	C1126 – C1145	2.03		
28	C1149 – C1169	2.03	1146 – 1270<br>hTG type-1 repeat 10	
29	C1181 – C1188	2.04		
30	C1190 – C1210	2.03		
31	C1215 – C1264	N.A.		C1215 modeled<br>C1264 not modeled
32	C1231 – C1245	2.03		Disulfide bond present in structure
33	C1249 – C1281	2.03		
34	C1306 – C1356	N.A.		C1306 modeled<br>C1356 not modeled
35	C1331 – C1347	2.03		Disulfide bond present in structure
36	C1440 – C1459	2.03	1456 – 1469<br>hTG type-2 repeat 1	Disulfide bond present in structure

37	C1462 – C1473	2.03	1456 – 1469 hTG type-2 repeat 1<br>1470 – 1486 hTG type-2 repeat 2	
38	C1476 – C1490	2.03	1470 – 1486 hTG type-2 repeat 2<br>1487 – 1503 hTG type-2 repeat 3	
39	C1493 – C1510	2.03	1487 – 1503 hTG type-2 repeat 3	
40	C1514 – C1523	2.03	1511 – 1565 hTG type-1 repeat 11	
41	C1543 – C1565	2.03		
42	C1603 – C1627**	2.03	1598 – 1723 hTG type-3a repeat 1	
43	C1607 – C1613	2.03		
44	C1639 – C1662**	N.A.		C1639 not modeled<br>C1662 modeled
45	C1724 – C1749	2.02	1724 – 1892 hTG type-3b repeat 1	Disulfide bond present in structure
46	C1728 – C1734	2.04		
47	C1733 – C1835**	N.A.		C1733 free<br>C1835 free and surface exposed
48	C1760 – C1777	2.03		Disulfide bond present in structure
49	C1893 – C1919	2.02	1893 – 1995 hTG type-3a repeat 2	
50	C1897 – C1904	2.02		
51	C1928 – C1939	2.03		
52	C1996 – C2024	2.03	1996 – 2129 hTG type-3b repeat 2	
53	C2000 – C2006	2.03		
54	C2005 – C2076	2.03		
55	C2035 – C2048	2.03		
56	C2130 – C2154	2.03	2130 – 2187 hTG type-3a repeat 3	
57	C2134 – C2140	2.04		
58	C2163 – C2172	2.03		
59	C2264 – C2281	2.02	2188 – 2727 ChEL	
60	C2442 – C2453**	2.03		
61	C2591 – C2715	2.04		

*… The amino acid numbering system presented is including signal peptide residues 1 – 19, following Uniprot: P01266 numbering (Date of download: 2020/04/20)

**... In the bovine TG structure (PDB ID 7N4Y), no disulfide bonds were observed between pairs of cysteine residues (bovine TG C63 – C70; C131 – C138; C330 – C336; C774 – C899; C1606 – C1630; C1642 – C1665; C1736 – C1837 and C2444 – C2455) corresponding to these pairs of hTG cysteine residues

Supplementary Table 4. Comparison of modification types and sites in hTG and bovine TG.

**Modification Site* and Type in hTG, structure This study**	**Modification Site* and Type in hTG, LC-MS This study**	**Modification Site# and Type in bovine TG structure, Kim et al. 2021**
Conserved, Not modeled	Y24, MIT, DIT, T_4	Y24, T_4
Conserved, Not modeled	Y108, No modification	Y108, dehydroalanine
Side chain density	Y149, MIT, DIT	Y149, dehydroalanine
Not conserved	Not conserved	Y1395, dehydroalanine
Not conserved	Not conserved	Y2041, DIT
Clear side chain density	Y2540, MIT, DIT, O-sulfonation	Y2542, dehydroalanine
Side chain density	Y2573, MIT, DIT	Y2575, T_4

*… The amino acid numbering system presented is including signal peptide residues 1 – 19, following Uniprot: P01266 numbering (Date of download: 2020/04/20)
… The amino acid numbering system presented includes signal peptide residues 1 – 19, following Uniprot: A0A4W2CHS8 numbering.

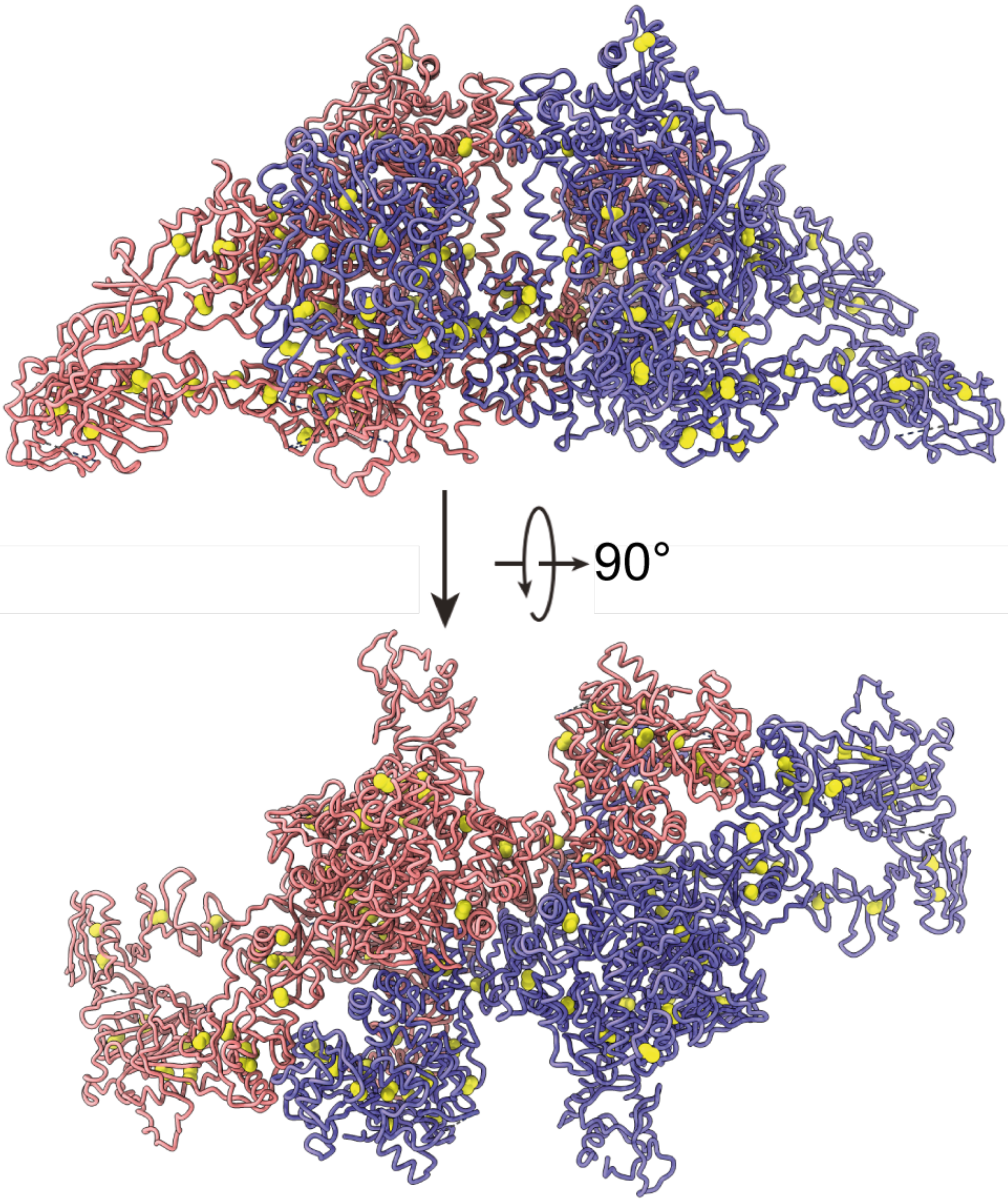

Supplementary Figure 1. Disulfide bridges (DSBs) in the hTG structure.
DSBs are represented as yellow spheres. The hTG monomers are visualized as purple and red ribbons.

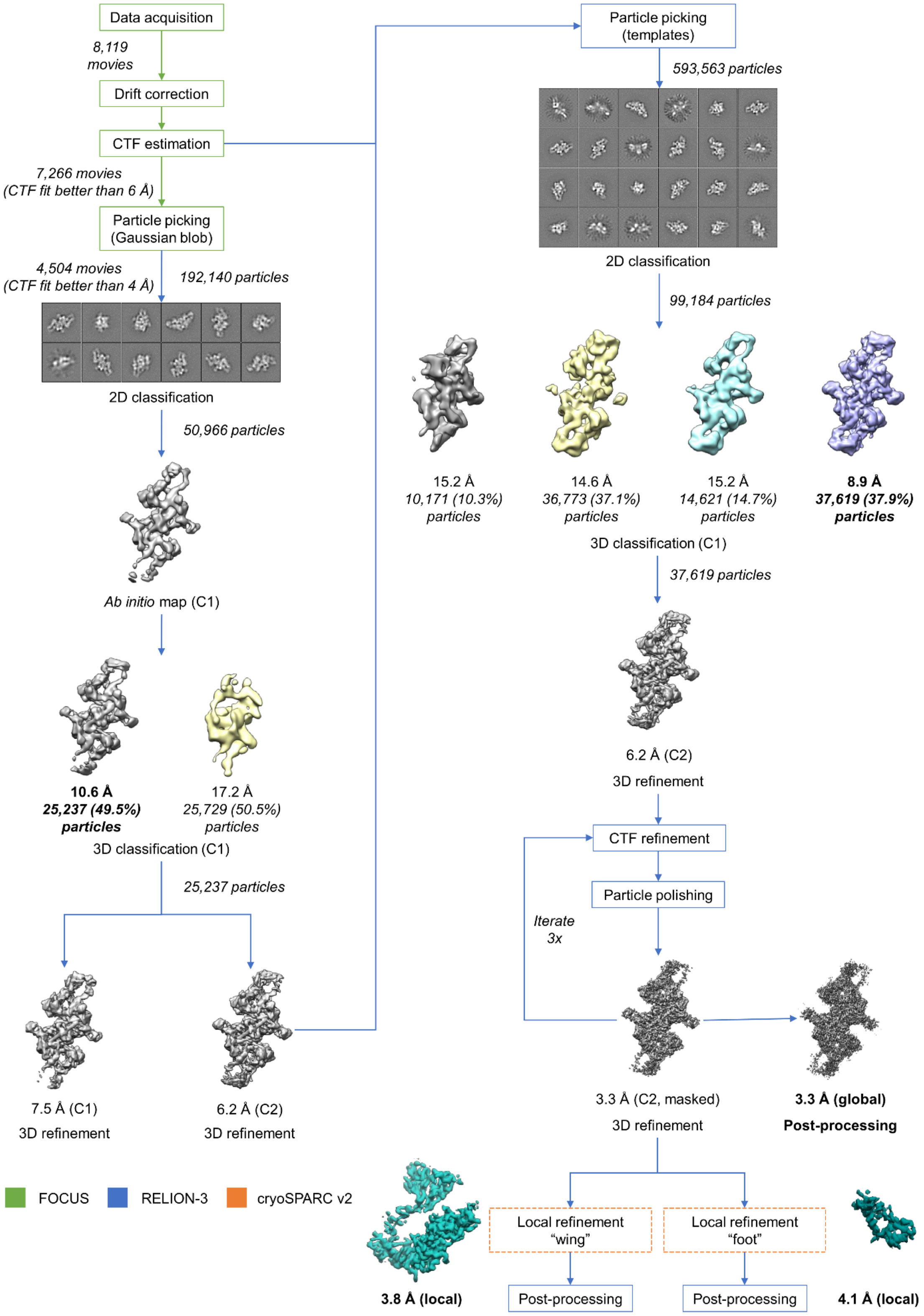

Supplementary Figure 2. Processing workflow of hTG cryo-EM dataset.
See also Methods for details.

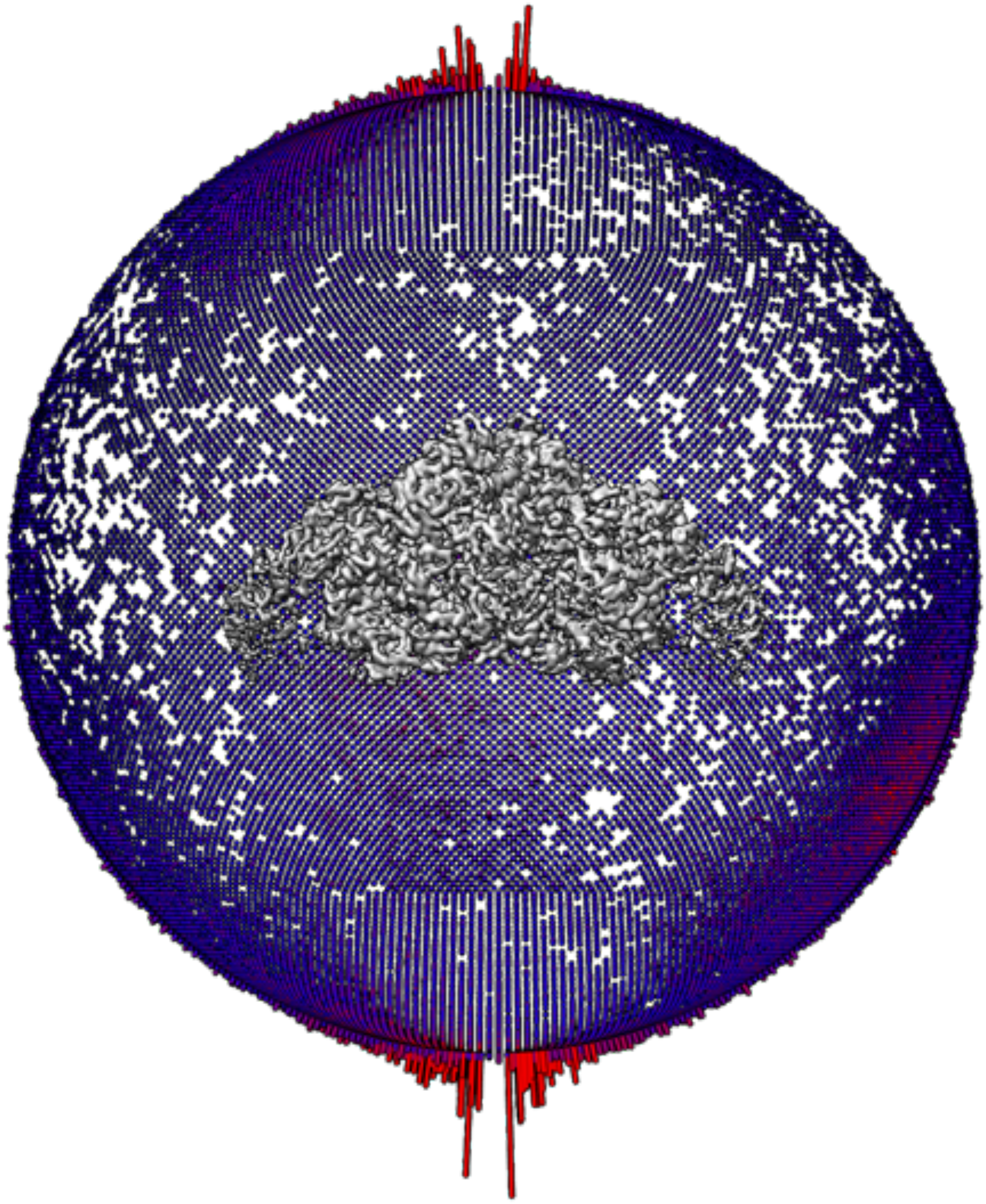

Supplementary Figure 3. Angular distribution plot,

The angular distribution plot is shown for the hTG consensus map.

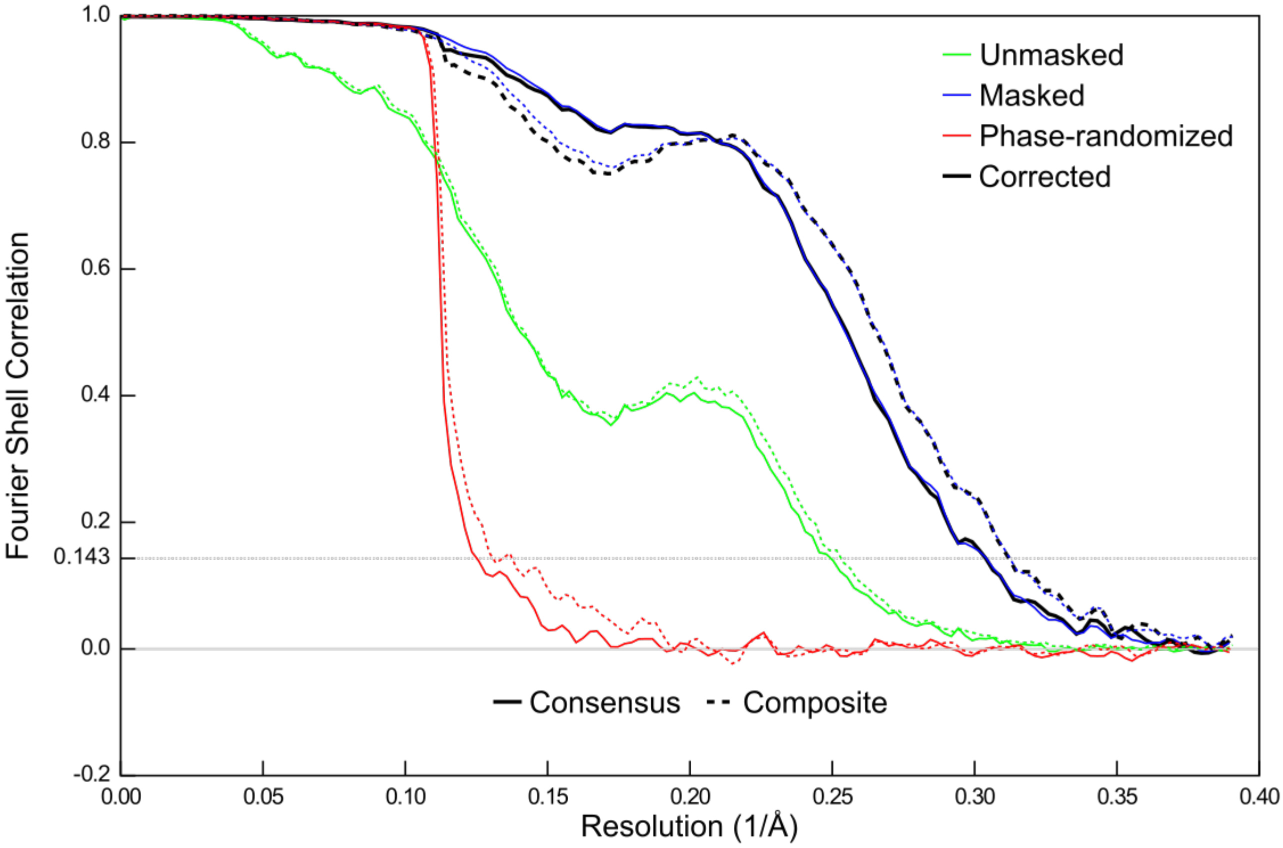

Supplementary Figure 4. Fourier shell correlation (FSC).

FSC curves for the hTG consensus and composite reconstructions.

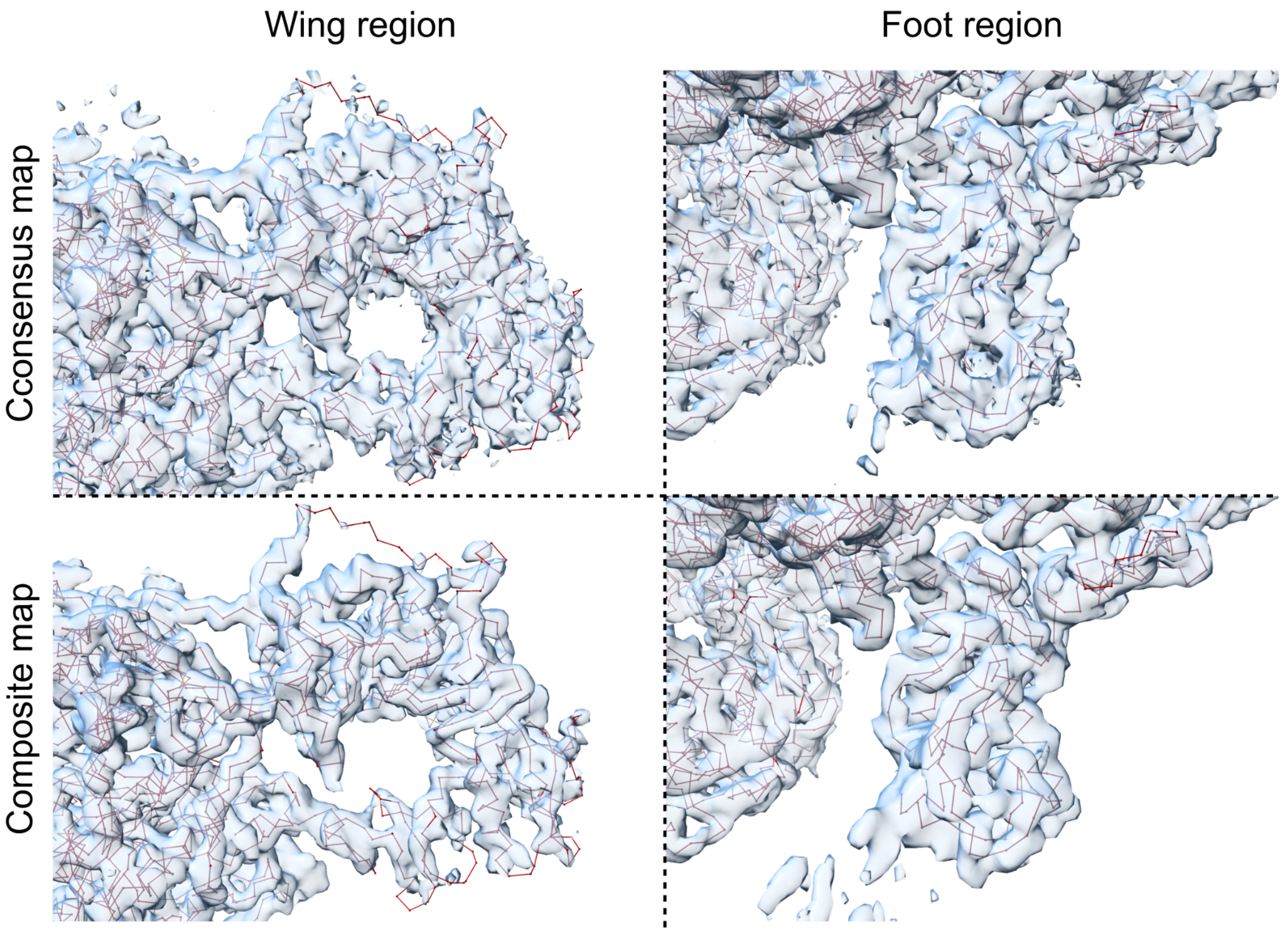

Supplementary Figure 5. Comparison of the “foot” and “wing” regions in the consensus map and in the composite (local refinement) maps.

Comparison is done at 7σ threshold levels. Note the foot local refinement was performed with signal-subtracted particles while the wing was not.

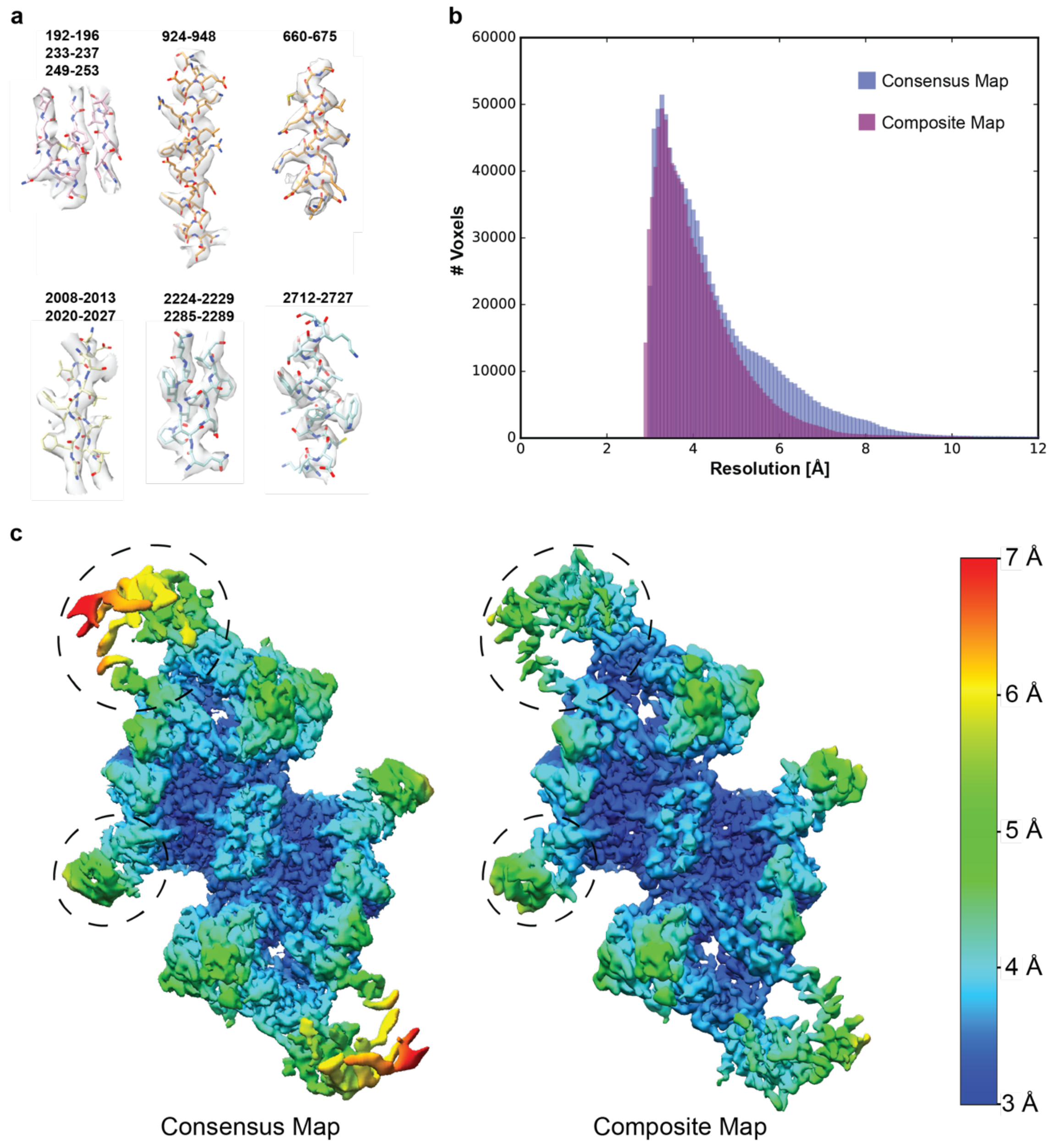

Supplementary Figure 6. Map quality.

(a) Selected fragments of the refined hTG atomic model shown inside the composite electron density map. **(b)** Local resolution histograms of the consensus and composite hTG maps. **(c)** Consensus and composite electron density maps colored by local resolution with flexible “wing” and “foot” regions in dashed ovals.

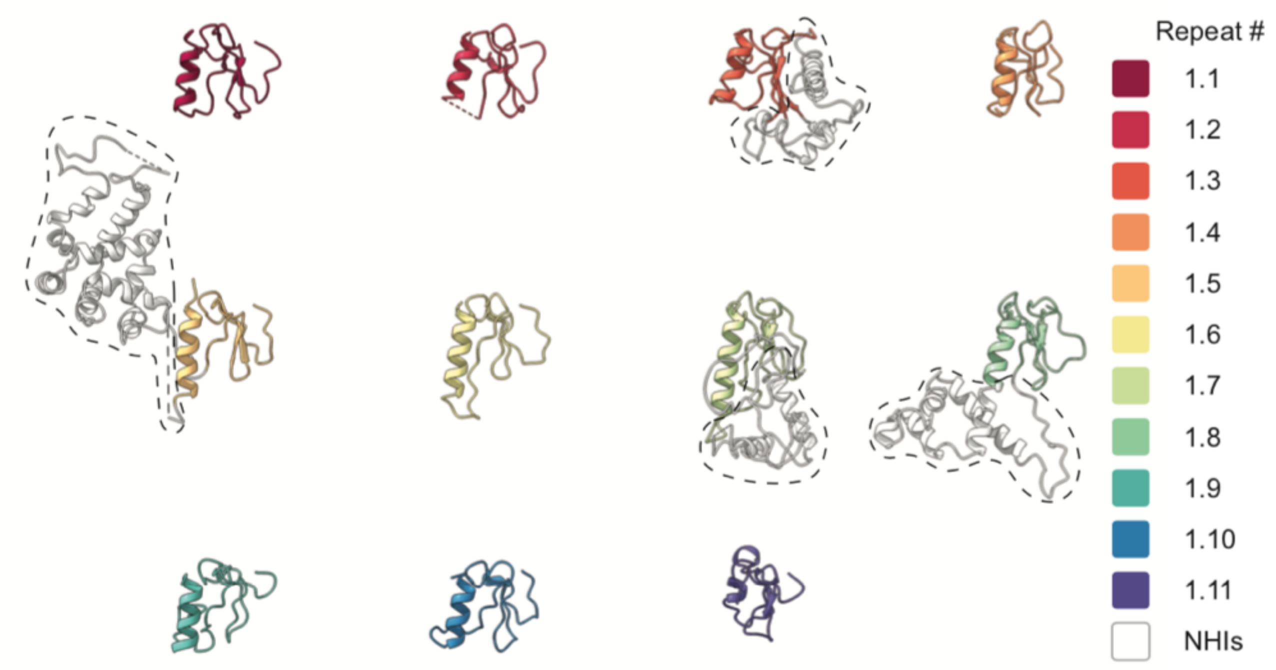

Supplementary Figure 7. Type 1 repeats.

Color code for each repeat on the right. NHIs are highlighted with a dashed line.

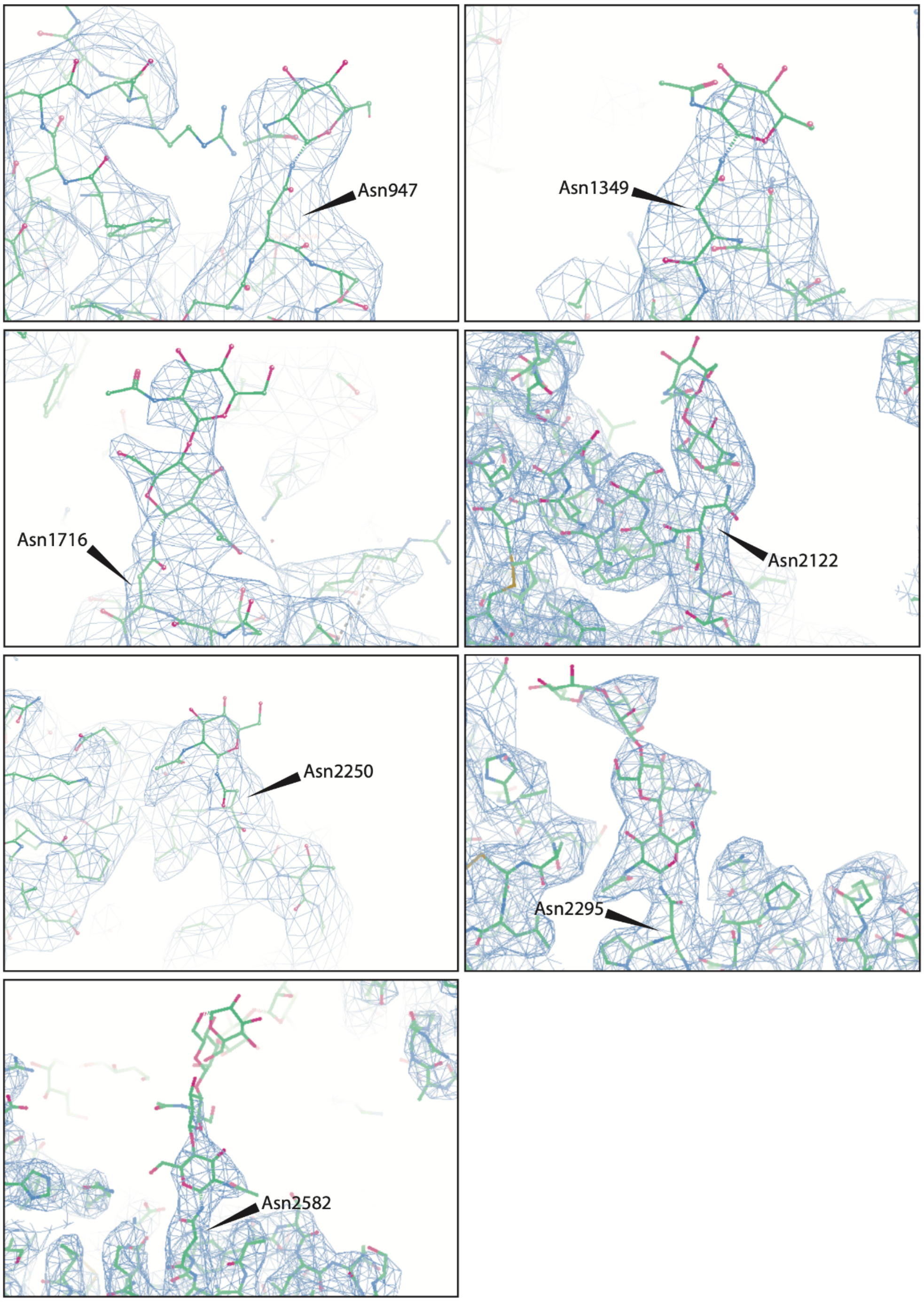

Supplementary Figure 8. Representation of modeled and MS-confirmed N-glycosylation sites inside the composite electron density map.

See also Table 1.

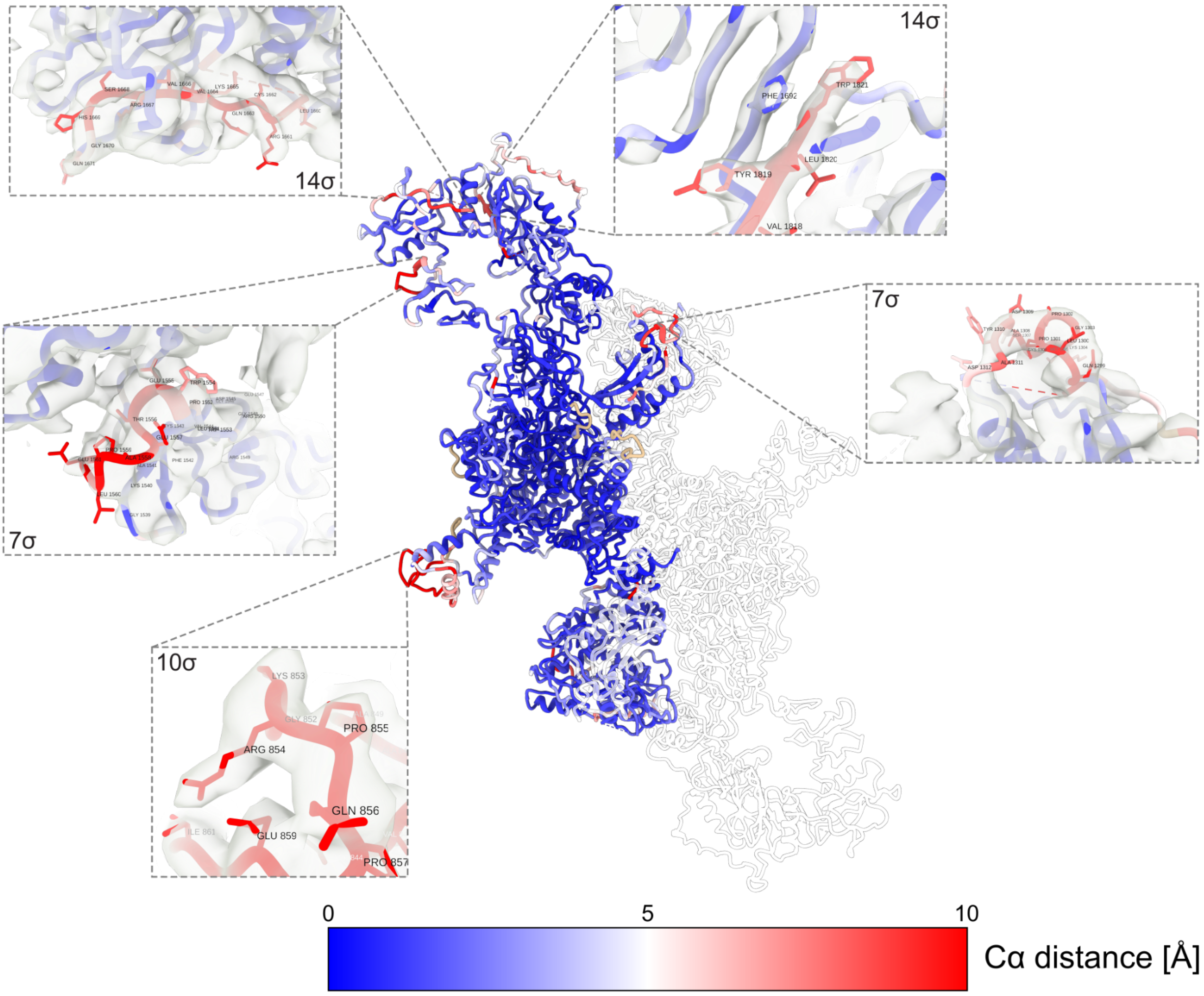

Supplementary Figure 9. Comparison of the hTG model obtained in this study with PDB ID 6SCJ (Coscia *et al.* 2020).

Chain A of the model is colored by the distance between Cα atoms after chain alignment and model superposition in Chimera. Loops shown in beige are not modelled in PDB ID 6SCJ. Insets show the fit into the cryo-EM density for selected areas of high RMSD between the two models, with the threshold level in standard deviations above the mean voxel value indicated. We refer the reader to Fig. S6 for comparison with the local resolution analysis of the map.
