# Supplementary material for: Cryo-EM structure of native human thyroglobulin
Source: Nat Commun. 2022 Jan 10;13:61. doi: 10.1038/s41467-021-27693-8 (PMC8748809; doi:10.1038/s41467-021-27693-8)
Supplement: Supplementary file 3 — Description of Additional Supplementary Files [file 41467_2021_27693_MOESM3_ESM.docx]

Description of Additional Supplementary Files

File name: Supplementary Movie 1

Description: Overview of human thyroglobulin structure.
