# Supplementary material for: Cryo-EM structure of native human thyroglobulin
Source: Nat Commun. 2022 Jan 10;13:61. doi: 10.1038/s41467-021-27693-8 (PMC8748809; doi:10.1038/s41467-021-27693-8)
Supplement: Supplementary file 5 — Reporting Summary [file 41467_2021_27693_MOESM5_ESM.pdf]

nature portfolio

Corresponding author(s): Nicholas Taylor, Henning Stahlberg

Last updated by author(s): Nov 19, 2021

Reporting Summary

Nature Portfolio wishes to improve the reproducibility of the work that we publish. This form provides structure for consistency and transparency in reporting. For further information on Nature Portfolio policies, see our Editorial Policies and the Editorial Policy Checklist.

Statistics

For all statistical analyses, confirm that the following items are present in the figure legend, table legend, main text, or Methods section.

n/a	Confirmed	
☒	☐	The exact sample size (*n*) for each experimental group/condition, given as a discrete number and unit of measurement
☒	☐	A statement on whether measurements were taken from distinct samples or whether the same sample was measured repeatedly
☒	☐	The statistical test(s) used AND whether they are one- or two-sided<br>*Only common tests should be described solely by name; describe more complex techniques in the Methods section.*
☒	☐	A description of all covariates tested
☒	☐	A description of any assumptions or corrections, such as tests of normality and adjustment for multiple comparisons
☐	☒	A full description of the statistical parameters including central tendency (e.g. means) or other basic estimates (e.g. regression coefficient) AND variation (e.g. standard deviation) or associated estimates of uncertainty (e.g. confidence intervals)
☒	☐	For null hypothesis testing, the test statistic (e.g. *F*, *t*, *r*) with confidence intervals, effect sizes, degrees of freedom and *P* value noted<br>*Give P values as exact values whenever suitable.*
☒	☐	For Bayesian analysis, information on the choice of priors and Markov chain Monte Carlo settings
☒	☐	For hierarchical and complex designs, identification of the appropriate level for tests and full reporting of outcomes
☒	☐	Estimates of effect sizes (e.g. Cohen's *d*, Pearson's *r*), indicating how they were calculated

Our web collection on statistics for biologists contains articles on many of the points above.

Software and code

Policy information about availability of computer code

Data collection	SerialEM v3.7 was used for data collection.
Data analysis	We used FOCUS v1.1.0, MotionCor2 v1, CTFFIND v4.1, Gautomatch v0.53, RELION v3, EMAN v2.12 , cryoSPARC v2, UCSF Chimera v1.14, UCSF ChimeraX v0.9 and v1.1, PHENIX v1.18 and Coot v0.9.2 for data processing and analysis, as described in the Methods.

For manuscripts utilizing custom algorithms or software that are central to the research but not yet described in published literature, software must be made available to editors and reviewers. We strongly encourage code deposition in a community repository (e.g. GitHub). See the Nature Portfolio guidelines for submitting code & software for further information.

Data

Policy information about availability of data

All manuscripts must include a data availability statement. This statement should provide the following information, where applicable:

- Accession codes, unique identifiers, or web links for publicly available datasets
- A description of any restrictions on data availability
- For clinical datasets or third party data, please ensure that the statement adheres to our policy

The EM map for the complete hTG molecule has been deposited in the EMDB under accession code EMD-12073 [https://www.ebi.ac.uk/pdbe/entry/emdb/EMD-12073]. Atomic coordinates for hTG have been deposited in the Protein Data Bank under the accession code PDB 7B75 [http://doi.org/10.2210/pdb7B75/pdb]. The mass spectrometry proteomics data has been deposited to the ProteomeXchange Consortium (http://proteomecentral.proteomexchange.org) via the PRIDE partner repository with the dataset identifier PXD029013 and 10.6019/PXD029013. LC-MS data used to generate tables and figures has been provided as a .xlsx Source Data file (SourceData_PTMs_Ionscore40_final.xlsx).

March 2021

Field-specific reporting

Please select the one below that is the best fit for your research. If you are not sure, read the appropriate sections before making your selection.

☒ Life sciences ☐ Behavioural & social sciences ☐ Ecological, evolutionary & environmental sciences

For a reference copy of the document with all sections, see nature.com/documents/nr-reporting-summary-flat.pdf

Life sciences study design

All studies must disclose on these points even when the disclosure is negative.

Sample size	8,119 micrographs were recorded in total. The final 3D reconstruction was obtained from 37,619 particles
Data exclusions	Micrographs were excluded based on the resolution of the CTF fit. Those with fit worse than 6 Å were excluded as they are unable to provide high-resolution information. 7,266 micrographs remained after exclusion as detailed in the Methods and Supplementary Figure 2. From the 593,563 particles picked from these micrographs. Particles assigned to "bad" classes in 2D and 3D classification, meaning they were damaged or suffered from preparation or imaging artefacts, were excluded. 37,619 remained up to the final reconstruction. Details are provided in the Methods.
Replication	See "Randomization" below as replication and randomization are closely related procedures in cryo-EM structure determination.
Randomization	The dataset was randomly split in two halves which were refined independently in RELION and cryoSPARC. Resolution was assessed by the Fourier shell correlation between the half-maps at the 0.143 threshold criteria, indicating the resolution which is reproducible from this dataset.
Blinding	Blinding is not applicable to this study. Particle assignment to half-sets and the corresponding resolution estimation were performed automatically by processing software packages.

Reporting for specific materials, systems and methods

We require information from authors about some types of materials, experimental systems and methods used in many studies. Here, indicate whether each material, system or method listed is relevant to your study. If you are not sure if a list item applies to your research, read the appropriate section before selecting a response.

Materials & experimental systems

n/a	Involved in the study	
☒	☐	Antibodies
☒	☐	Eukaryotic cell lines
☒	☐	Palaeontology and archaeology
☒	☐	Animals and other organisms
☒	☐	Human research participants
☒	☐	Clinical data
☒	☐	Dual use research of concern

Methods

n/a	Involved in the study	
☒	☐	ChIP-seq
☒	☐	Flow cytometry
☒	☐	MRI-based neuroimaging
